# The effect of two types of ankle orthoses on the repetitive rebound jump performance

**DOI:** 10.1186/s13102-022-00478-2

**Published:** 2022-05-16

**Authors:** Masanori Morikawa, Noriaki Maeda, Makoto Komiya, Toshiki Kobayashi, Yukio Urabe

**Affiliations:** 1 Division of Sport Rehabilitation, Graduate School of Biomedical and Health Sciences, Hiroshima University, 1-2-3 Kasumi, Minami-ku, Hiroshima, 734-8553 Japan; 2 Department of Preventive Gerontology, Center for Gerontology and Social Science, National Center for Geriatrics and Gerontology, Obu, Japan; 3 Department of Biomedical Engineering, Faculty of Engineering, The Hong Kong Polytechnic University, 11 Yuk Choi Road, Hung Hom, Hong Kong, China

**Keywords:** Ankle lateral ligament, Sports performance, Ankle brace, Ankle support, Ankle orthotics

## Abstract

**Background:**

Ankle orthotics decreases the maximal vertical jump height. It is essential to maximize jump height and minimize ground contact time during athletic performance. However, the effect of ankle orthotics on athletic performance has not been reported. We aimed to investigate the effect of ankle orthotics on squat jump (SJ), countermovement jump (CMJ), and repetitive rebound jump (RJ) performance.

**Methods:**

Twenty healthy volunteers performed SJ, CMJ, repetitive RJ under no-orthosis and two orthotic conditions (orthosis 1 and orthosis 2). During SJ and CMJ, we measured the vertical ground reaction force and calculated the following parameters: jump height, peak vertical ground reaction force, rate of force development, net vertical impulse, and peak power. During repetitive RJ, the jump height, contact time, and RJ index were measured. A two-dimensional motion analysis was used to quantify the ankle range of motion in the sagittal plane during SJ, CMJ, and repetitive RJ.

**Results:**

Multivariate analysis of variance and the post hoc test showed orthosis 2 significantly decreased in the vertical jump height (*p* = 0.003), peak power (*p* = 0.007), and maximum plantarflexion and dorsiflexion angles (*p* < 0.001) during SJ Ankle orthoses 1 and 2 did not influence to the RJ performance compared to those using the no-orthosis condition. Additionally, orthosis 2 significantly decreased the jump height at the end of repetitive RJ (*p* = 0.046).

**Conclusions:**

These results suggest that ankle orthosis do not affect average RJ performance but should be considered when performing repetitive jumps frequently.

## Background

Ankle orthotics is a common measure for preventing ankle sprain. Meta-analyses have shown that ankle orthotics is effective for the primary and secondary prevention of acute ankle injuries among athletes [1]. Nevertheless, ankle orthotics may have a negative impact on athlete performance in activities such as sprinting [2], cutting [3], jump landing [4, 5], and vertical jumping [2]. Furthermore, there is a consensus that ankle orthotics reduces the vertical jump height [6–12]. Also, the features of ankle orthoses should be considered as they affect jumping performance [8]. Ankle orthoses are biomechanically designed using a wide variety of materials to control, limit, and immobilize the motion for ankle joint. This effect of jump performance may be due to a decrease in the ankle range of motion (ROM) in the sagittal plane [10, 11], rectus femoris and calf muscle activity [8, 9, 11], or vertical ground reaction force (VGRF) [8, 12].

However, since the ankle orthosis-related decrease in jump height is small (0.013 m) [10], it is controversial whether ankle orthotics influences vertical jump performance during practice or competition. Most previous studies focused on the maximum jump height for static vertical jumps such as squat jump (SJ) [8–10] or countermovement jump (CMJ) [6–8, 11] not for repetitive rebound jump. Reactive strength may be used to assess the athlete’s ability to attain maximum jump height and minimum ground contact time during repetitive rebound jump (RJ) [13]. It also indicates an athlete’s ability to rapidly generate force under a high eccentric load [14]. Jump height, ground contact time, and reactive jump index (ratio of jump height and ground contact time) were used as parameters in previous studies to evaluate jump performance in the sports field [15]. However, no study has investigated whether the use of ankle orthotics decreases repetitive RJ performance.

Therefore, we aimed to investigate the influence of ankle orthotics on SJ, CMJ, and repetitive RJ performance, and the relationship between jump performance and dorsi-plantarflexion ROM in healthy adults by using different type of ankle orthosis. We hypothesized that 1) ankle orthoses decrease SJ and CMJ performance (as in previous studies [6–11]), while do not affect RJ performance, but 2) the effect on jumping performance more pronounced as the degree of plantar dorsiflexion range-of-motion restriction increases.

## Methods

### Participants

Twenty recreationally active volunteers (15 men and 5 women) agreed to participate in the study (mean ± standard deviation [SD] of age, body height, weight, and body mass index: 23.9 ± 2.5 years, 168.4 ± 8.41 cm, 61.8 ± 12.5 kg, and 21.6 ± 3.1 kg/m^2^, respectively) after being informed about this study protocol. The study protocol was approved by the Ethics Committee for epidemiology of Hiroshima University (approval number: E-2268). All subjects provided informed consent for their participation in the study. “Recreationally active” was defined as participation in at least 150 min of moderate activity per week for at least 6 months prior to the study [16]. Participants were experienced in athletics, basketball, baseball, classical ballet, badminton, football, tennis, swimming, or volleyball. We excluded participants with lower extremity injury and symptoms, previous lower extremity surgery prior to the study, vestibular disease, or neurological impairments.

## Study design and procedures

**Fig. 1:**
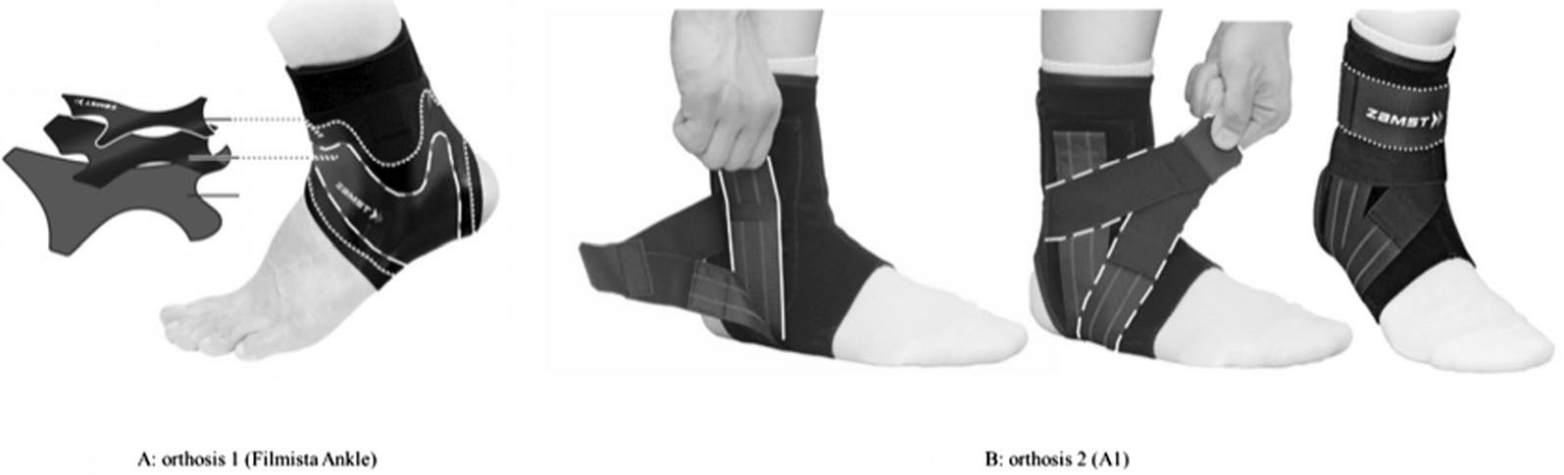
Ankle orthotics used in this study. **A**: The long-dotted line indicates the hard film in the surface layer. The short-dotted line indicates the soft film in the middle layer. **B**: The solid line indicates the stirrup strap. The long-dotted line indicates the biceps strap. The short-dotted line indicates the strap for distal tibiofibular joints

This study used a laboratory-based and repeated-measures test design. To determine the influence of ankle orthotics on jump performance and dorsi-plantarflexion ROM, we used the following experimental protocol with participants in a barefoot condition (no-orthosis) and two orthotics conditions (orthosis 1 and orthosis 2) with different restrictions on dorsi-plantarflexion. Filmista (Nippon Sigmax, Japan) and A1 (Nippon Sigmax, Japan) orthotics were used for orthosis 1 and 2 conditions, respectively. Participants wore correctly sized orthotics on both ankles. A certified orthotist instructed participants on how to wear the orthotics using demonstrations. Figure 1 shows the composition of the orthotics used in this study. Orthosis 1 consists of three thin layers (Fig. 1A). It has two surfaces made of soft and hard urethane films with different elasticities. The hard film is found in the middle layer and is designed to limit excessive ankle inversion with softly restricting the ROM of the ankle joint in the sagittal plane. Orthosis 2 consists of three different straps on the fabric, covering the ankle joint with medial and lateral stays (Fig. 1B). Stirrup, biceps, and distal tibiofibular joint straps are applied to prevent excessive ankle inversion, based on the medical taping concept. These ankle orthoses were made by the same manufacturer; however, orthosis 1 had less restriction on ankle inversion than orthosis 2. Participants successively repeated the same jump exercises under the three abovementioned conditions following a randomized order. A jump session was defined as a period during which a participant performed static jumps and RJ under one of the above conditions. All parameters were measured over a 3-day period for each participant. Participants completed a jump session under one condition per day, with a minimum of 24 h of rest between each jump session.

## Vertical jump performance tests

Participants performed a 5-min warm-up exercise before undergoing the vertical jump performance tests. They received explanations on how to perform SJ, CMJ, and RJ and practiced these vertical jumps. To perform the SJ, they started by folding their hands and squatting with their hips and knees flexed to approximately 45 $^\circ$ and their feet placed a shoulder width apart on a force plate (Technology Service, Nagano, Japan). After 1–2 s, the examiner instructed the participants to jump vertically and forcefully. To perform the CMJ, they began by folding their hands and standing upright with their feet placed approximately a shoulder width apart on the force plate. After 1–2 s, they rapidly descended into a 45 $^\circ$ semi-squat position and jumped vertically with maximum effort. Five sets, each of SJ and CMJ, were performed. Participants took as many breaks as needed between each set to avoid fatigue.

The repetitive RJ performance was assessed using the Optojump™ system (Microgate, Bolzano, Italy), consisting of two infrared photocell bars with one bar acting as a transmitter unit containing 96 light-emitting diodes positioned 3 mm above the ground, and the other bar acting as a receiver unit. Participants were instructed to keep their hands on their hips to avoid upper-body interference, jump, and land on the same spot, with legs extended then flexed, while looking ahead. They were also instructed to maximize the jump height and minimize the ground contact time. In a previous study, this method of RJ assessment achieved interday reliability [17]. When a participant performed the repetitive RJ within a parallel bar configuration, the light from the light-emitting diode was interrupted by the participant’s foot during the jump, triggering the timer in the unit and recording the interruption with a sampling frequency of 1000 Hz. Two sets of seven repetitive RJs were performed with intermittent 5-min resting periods.

Before all protocols were performed, participants practiced three times each for SJ and CMJ and one set (six repetitive jumps) for RJ.

## Analysis of the vertical jump performance

SJ and CMJ performances were analyzed using the VGRF, recorded by a force plate with a sampling frequency of 1000 Hz, using a zero-lag, fourth-order, low-pass Butterworth filter with a cutoff frequency of 20 Hz. Based on previous studies [18], jump height [cm], rate of force development (RFD) [N/s], vertical impulse [Ns], peak power [W], and maximum VGRF (VGRFmax) [N] were calculated using MATLAB (R2020b, Math Works GK, Tokyo, Japan). The jump height was estimated as follows: jump height = (1/2 × Tair × g)^2^ × (2 g)^−1^. Tair represents the flight time [s] from the force record on the force plate, and g the acceleration due to gravity (9.81 m/s^2^). The RFD was calculated as: RFD = (VGRFmax–minimum VGRF [VGRFmin]) / Δt1, where Δt1 indicates the change in time [s] between 20 and 80% of the total time from the VGRFmin to the VGRFmax. VGRFmin was defined as the lowest value of the VGRF during the contact phase before increasing to VGRFmax. VGRFmax was defined as the peak of the VGRF occurring for the first time if two peaks were applicable. The net vertical impulse was calculated as VGRF × Δt2 body weight × Δt2, where Δ2 indicates the change in time [s] from the point at which the VGRF equated with the body weight to the point at which VGRF fell below the body weight. Peak mechanical power was calculated from the vertical jump height and body weight as (60.7 × jump height [cm]) + (45.3 × body height [cm]). The RFD and VGRFmax were normalized using the body weight [kg] to calculate the relative RFD and relative VGRFmax.

Optojump™ proprietary software (Optojump™ Next software, version 1.9.9.0, Bolzano, Italy) was used to automatically calculate RJ performance variables (jump height [cm], contact time [s], and RJ index (RJ index) [m/s]). The RJ index was estimated as: RJ index = 1/8 × g × Tair^2^ / contact time. Of the seven repetitive jumps in the second set, the second, third, fourth, and fifth jumps were included in the analysis. The individual performance variables per RJ (second, third, fourth, fifth, and sixth jumps) and their means were used for further statistical analysis.

## Analysis of the sagittal ankle joint motion

**Fig. 2:**
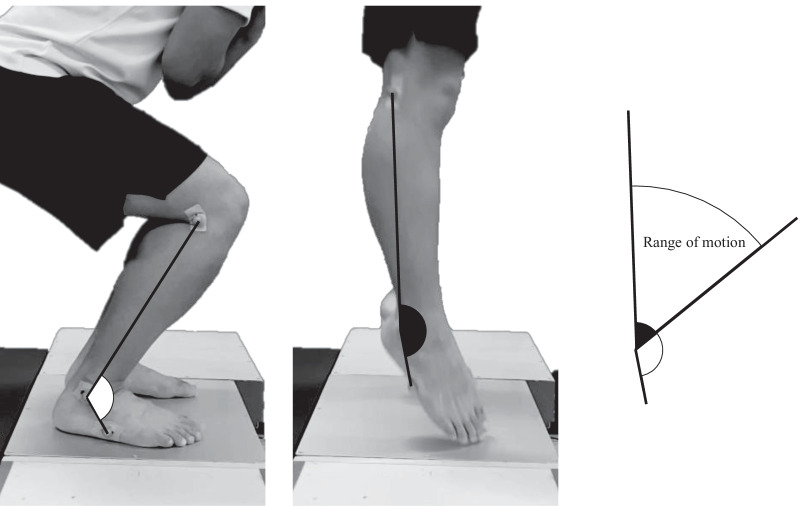
Video analysis of maximum dorsiflexion angle (**A**), plantarflexion at toe-off (**B**), and dorsi-plantarflexion ROM (**C**). ROM, range of motion

Two-dimensional motion analysis for sagittal ankle joint motion was performed simultaneously with the vertical performance test parameters (SJ, CMJ, and RJ). Three reflective markers were placed on the dominant leg on the lateral aspect of the tibial plateau, lateral malleolus, and lateral aspect of the base of the fifth metatarsal [10]. The markers were applied by the same examiner. A video camera was positioned at a distance of 1.5 m perpendicular to the edge of the force plate or OptoJump™ device to capture the trajectory of the marker from the sagittal plane with a sampling frequency of 240 Hz. ImageJ software (National Institutes of Health, Maryland, USA) was used for the analysis. The maximum dorsiflexion angle, plantarflexion angle at toe-off, and dorsi-plantarflexion ROM were calculated by connecting three points [10], as shown in Fig. 2. The ROM was defined as the difference between the plantarflexion angle at toe-off and the maximum dorsiflexion angle. For each joint angle, the mean angle from the five jumps was used as the representative value. Additionally, the individual angle of the second set of RJ was used for further analysis.

## Statistical analysis

A one-factor (type of orthosis: no-orthosis, orthosis 1, orthosis 2) repeated-measures multivariate analysis of variance (MANOVA) was used to determine the influence of ankle orthotics on the variables of jump performance and ankle ROM. MANOVA was conducted for each type of jump (SJ, CMJ, RJ). A univariate analysis of variance (ANOVA) and Tukey method for pairwise comparisons were conducted on any significant findings.

To determine the difference in the effect of ankle orthosis use on each RJ-related variable (jump height, contact time, and RJ index) and the angle for sagittal ankle motion by the number of RJs, a two-factor (type of orthosis: no-orthosis, orthosis 1, orthosis 2 × number of RJ: second, third, fourth, fifth, and sixth) repeated-measures MANOVA was initially conducted. As a follow-up analysis, a two-factor ANOVA was conducted on any significant findings. If the main or interaction effect was observed, the Tukey method of pairwise comparisons was also performed. Another repeated-measure analysis of variance and pairwise comparisons was conducted to determine the difference in the number of RJ (second, third, fourth, fifth, and sixth) regard to jump height, contact time, and RJ index respectively.

A post hoc power analysis was performed to calculate the statistical power for primary outcome using G*Power 3.1.9.2 [19]. A significant level was set at P value of < 0.05. All statistical tests were performed using the SPSS® software.

## Results

Table 1 summarizes the mean ± SD of the measurement variables in this study and their levels of statistical significance. MANOVA showed a significant difference in the vertical jump performance only during SJ performance (*p* = 0.021) under orthotic conditions; the vertical jump performances were not significantly different for participants who performed CMJ (*p* = 0.118) and RJ (*p* = 0.391). The mean jump height, peak power, and angle of sagittal ankle motion during SJ performance systematically decreased in the following order: no-orthosis, orthosis 1, and orthosis 2 conditions. Pairwise comparisons after univariate analysis showed a significant decrease in the vertical jump height (*p* = 0.003), peak power (*p* = 0.007), and sagittal ankle ROM (*p* < 0.001) during SJ performance using orthosis 2 compared to the same parameters measured under no-orthosis conditions.

**Table 1:** Mean ± standard deviation of measurement variables during squat, counter movement, and rebound jumps under three conditions and their level of statistical significance

	No-orthosis	orthosis 1	orthosis 2	*p*-value^a^	effect size (f)	significance^b^
Squat jump						
Jump height, cm	26.6 ± 5.2	26.5 ± 5.3	25.3 ± 5.5	0.003	0.594	no-orthosis, 1 vs 2
RFD, N/s	2602 ± 1158	2798 ± 1182	2682 ± 1203	0.633	0.157	
rRFD, N/kg/s	42.9 ± 20.3	46.3 ± 19.4	45.1 ± 23.3	0.643	0.153	
Vertical impulse, Ns	166.1 ± 49.2	162.5 ± 44.9	168.4 ± 54.6	0.417	0.217	
PeakPower, W	2356 ± 706	2339 ± 676	2274 ± 687	0.007	0.543	no-orthosis vs 2
VGRFmax, N	1421 ± 308	1424 ± 277	1428 ± 286	0.939	0.055	
rVGRFmax, N/kg	23.0 ± 2.2	23.1 ± 2.1	23.2 ± 2.3	0.831	0.101	
Maximum dorsiflexion angle, degree	120.6 ± 5.5	125.4 ± 6.8	127.3 ± 5.0	< 0.001	0.811	no-orthosis vs 1, 2
Plantarflexion angle at toe off, degree	171.2 ± 5.8	167.1 ± 6.9	163.3 ± 4.5	< 0.001	1.077	no-orthosis vs 1 vs 2
ROM, degree	50.3 ± 4.7	41.6 ± 5.4	35.8 ± 3.9	< 0.001	2.409	no-orthosis vs 1 vs 2

Countermovement jump						
Jump height, cm	28.3 ± 5.6	28.3 ± 5.5	27.8 ± 5.9	N/A	N/A	
RFD, N/s	3277 ± 1878	2971 ± 1615	3062 ± 1877	N/A	N/A	
rRFD, N/kg/s	53.9 ± 29.2	49.2 ± 26.4	50.7 ± 30.5	N/A	N/A	
Vertical impulse, Ns	157.4 ± 33.4	141.2 ± 37.5	154.8 ± 38.8	N/A	N/A	
PeakPower, W	2459 ± 742	2448 ± 705	2423 ± 718	N/A	N/A	
VGRFmax, N	1391 ± 299	1356 ± 271	1378 ± 284	N/A	N/A	
rVGRFmax, N/kg	22.5 ± 2.0	22.0 ± 2.1	22.4 ± 2.6	N/A	N/A	
Maximum dorsiflexion angle, degree	117.6 ± 7.6	122.7 ± 6.7	124.5 ± 6.1	** < 0.001**	0.703	no-orthosis vs 1, 2
Plantarflexion angle at toe off, degree	170.9 ± 6.3	167.3 ± 6.7	163.9 ± 4.6	** < 0.001**	0.883	no-orthosis vs 1 vs 2
ROM, degree	53.3 ± 5.5	44.5 ± 5.6	39.3 ± 3.8	** < 0.001**	1.940	no-orthosis vs 1 vs 2

Repetitive rebound jump						
Jump height, cm	26.3 ± 6.6	26.0 ± 7.2	25.1 ± 6.1	N/A	N/A	
contact time, s	0.22 ± 0.04	0.22 ± 0.04	0.24 ± 0.08	N/A	N/A	
Rebound jump index, m/s	1.26 ± 0.34	1.24 ± 0.31	1.18 ± 0.31	N/A	N/A	
Maximum dorsiflexion angle, degree	124.9 ± 8.4	128.8 ± 7.4	131.4 ± 6.8	** < 0.001**	1.784	no-orthosis vs 1, 2
Plantarflexion angle at toe off, degree	170.0 ± 7.6	165.9 ± 7.4	163.8 ± 4.2	** < 0.001**	1.652	no-orthosis vs 1, 2
Range of motion, degree	44.9 ± 7.5	36.8 ± 6.0	32.4 ± 5.4	** < 0.001**	1.825	no-orthosis vs 1 vs 2

a: *P*-values indicate the results of the statistical analysis of pairwise comparisons. *RFD* Rate of force development; *rRFD* Relative rate of force development; *VGRFmax* Maximum vertical ground reaction force; *rVGRFmax* Relative maximum vertical ground reaction force; *ROM* range of motion. b: This significance was obtained from the multiple comparison with the Bonferroni correction and described the large and small relationships that were less than *p* < 0.05. *P*-values were calculated using multivariate analysis of variance with repeated measures (no-orthosis, orthosis 1, and orthosis 2).

**Fig. 3:**
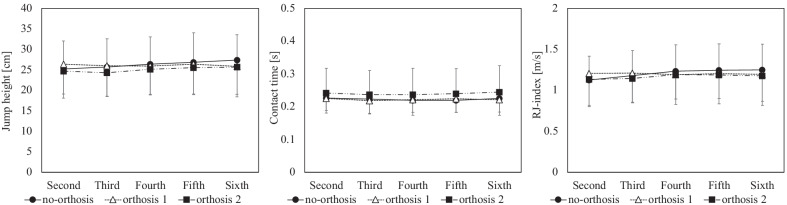
Impact of two types of ankle orthotics on repetitive rebound jump performance. Two-factor (condition: no-orthosis, orthosis 1, orthosis 2 × number of rebound jumps: second, third, fourth, fifth, sixth) repeated-measures multivariate analysis of variance showed significant difference in repetitive rebound jump performance (*f* = 7.020, *p* < 0.001). One-factor (condition: no-orthosis, orthosis 1, orthosis 2) repeated-measures analysis of variance and pairwise comparisons revealed a significant decrease in jump height in orthosis 2 compared to the no-orthosis condition. Another repeated-measure analysis of variance and pairwise comparisons showed significant difference between second and sixth jump heights only in no-orthosis. * = statistically significant difference (*p* < 0.05). † = statistically significant difference (*p* < 0.01)

**Fig. 4:**
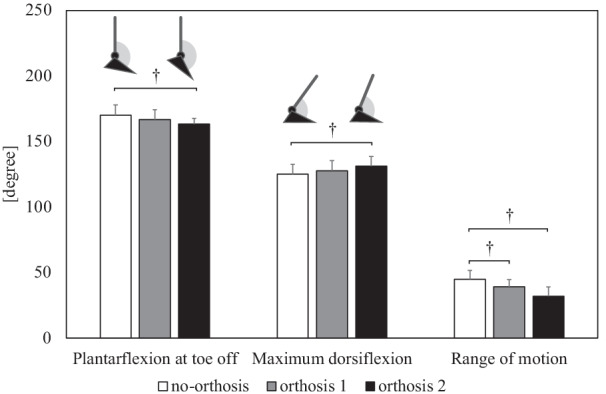
Influence of two types of ankle orthotics on sagittal angle of ankle joint at the sixth rebound jump. † = statistically significant difference (*p* < 0.01)

Figure 3 shows the temporal changes in jump height, contact time, and RJ index during repetitive RJ, depending on the orthosis condition. The jump height significantly decreased only at the sixth RJ under the orthosis 2 condition compared to that under the no-orthosis condition. In non-orthosis condition, the second jump height was less than the sixth jump height. At the sixth RJ, as shown in Fig. 4, the angle of maximum dorsiflexion, plantarflexion at toe-off, and their ROMs were restricted under the orthosis 2 condition, which was a significant change compared to that under the no-orthosis condition (*p* < 0.001).

The post hoc power analysis showed the power of 92.2% with an effect size (f) of 0.740 for the primary outcome (RJ-index).

## Discussion

This study aimed to investigate the effect of ankle orthotics with different degrees of restriction of the ankle ROM in the sagittal plane, particularly on repetitive RJ performance. Ankle orthoses did not influence on repetitive RJ performance (contact time, jump height, RJ index). On the other hand, when analyzed by number of repetitive RJs, we observed unusual jump performance: jump heights were higher in the sixth than in the second in the no-orthosis condition, but there was no significant difference between any number of jumps in both of orthosis 1 and 2 conditions. Furthermore, the sixth jump height in the orthosis 2 condition was significantly lower than in the no-orthosis condition. These results suggest that ankle orthosis does not affect average RJ performance but should be considered when performing repetitive jumps frequently.

The repetitive jump heights increased with the number of jumps under the no-orthosis condition, but not under orthotic conditions; the difference in jump height was significant at the end of RJ. A previous study reported that the stretch–shortening cycle, enabling the production of high muscle forces and elastic energy storage [20], is a key muscle ability during repetitive RJ [21] because it allows for an explosive release of stored energy for subsequent jumps. Thus, ankle orthotics may interfere with this normal function during repetitive RJ. Additionally, the sagittal ankle ROM was significantly restricted in the orthosis 2 condition during repetitive RJ. Importantly, maximum dorsiflexion occurs during the landing phase, which coincides with the critical period of force storage and may affect the energy released for the following jump phase.

The jump height, peak power, and ankle ROM were systematically decreased in SJ in the order of no-orthosis, orthosis 1, and orthosis 2 conditions. Moreover, a significant difference was recorded in these parameters between the orthosis 2 and no-orthosis condition. Henderson et al. [9] reported that two ankle orthotics with similar restrictive functions decreased jump height and ankle ROM in the sagittal plane during SJ; these parameters did not significantly change under the no-orthosis condition in this previous study. Another study found a decrease in peak power and jump height during SJ [22]. These studies indicate that restricted ankle ROM in the sagittal plane caused by ankle orthotics is associated with reduced jump height as a result of decreased peak power; these results were corroborated by our study findings. In contrast, the ankle orthotics did not significantly decrease jump height in CMJ and RJ. Although ankle orthotics tended to decrease jump height, the negative effect of ankle orthotics may relatively decrease during complex movements. Although additional research including a more detailed examination of kinematics and kinetics is warranted, it is possible that the sagittal range of motion restriction caused by the wearing orthosis prevented normal lower extremity power exertion, leading to a more pronounced loss of jumping performance in SJ, which requires relatively pure lower extremity function compared to CMJ and RJ.

The present study has some limitations. First, it is difficult to generalize our results to other types of ankle orthotics. In addition to the soft ankle orthosis used in this study, a semi-rigid ankle orthosis may limit joint motion by a greater degree. However, the semi-rigid ankle orthosis was not used in this study, and it is not possible to present the effect of the type of ankle orthosis on repetitive RJ performance. Second, we did not recruit athletes with ankle sprains or chronic ankle instability; thus, further studies including these athletes may be required, although ankle orthosis is also used for the primary prevention of ankle sprains. Third, this study did not control for the presence or frequency of ankle brace use, so that different results may be obtained in athletes who require ankle braces. Fourth, the experiment was conducted with socks and shoes removed, the effect of ankle orthoses should be considered for each sporting situation. Fifth, although the certified orthotist fitted all participants with their ankle orthosis, it was unable to objectively standardize the degree of skin compression caused by the ankle orthosis. Sixth, this study did not measure the ankle joint motion on the frontal plane (inversion and eversion). Therefore, comprehensive measurements for ankle joint motions from the multiple planes are needed to explore findings related to ankle sprains. Seventh, this study did not assess the lower extremity range of motion. Therefore, it was not possible to consider differences in lower extremity range of motion between participants. Eighth, this study recruited the participants regardless of a kind of sports so that the modifying effect of a kind of sports needs to be investigated.

## Conclusions

The orthosis 2, which relatively restricted the range of plantar-dorsiflexion motion among orthotic condition, decreased the jumping height in SJ. However, CMJ and repetitive RJ, which required more complex jumping performance, were not influenced by any ankle orthoses use. On the other hands, when analyzed by number of repetitive RJs, jump height increased significantly with each repetition in the no-orthosis condition, but did not change in the orthosis 1 and 2 conditions. In addition, in the sixth jump height, orthosis 2 was significantly lower than no-orthosis. Therefore, this study indicates that ankle orthosis does not affect average RJ performance but should be considered when performing repetitive jumps frequently.

## Data Availability

The datasets generated and/or analysed during the current study are not publicly available due to the ethical consideration but are available from the corresponding author on reasonable request.

## Abbreviations

ANOVA: Analysis of variance
CMJ: Countermovement jump
MANOVA: Multivariate analysis of variance
RFD: Rate of force development
RJ: Rebound jump
ROM: Range of motion
SD: Standard deviation
SJ: Squat jump
VGRF: Vertical ground reaction force
VGRFmax: Maximum VGRF
VGRFmin: Minimum VGRF
RJ: Index
